# PHF20L1: An Epigenetic Regulator in Cancer and Beyond

**DOI:** 10.3390/biom15071048

**Published:** 2025-07-18

**Authors:** Yishan Wang, Qin Hu, Haixia Zhao, Lulu Zeng, Zhongwei Zhao, Xia Li, Qiaoyou Weng, Yang Yang, Minjiang Chen, Jiansong Ji, Rongfang Qiu

**Affiliations:** 1 Zhejiang Key Laboratory of Imaging and Interventional Medicine, The Fifth Affliated Hospital of Wenzhou Medical University, Lishui 323000, China; wangyishan@wmu.edu.cn (Y.W.); huqin@wmu.edu.cn (Q.H.); zhaohaixia@wmu.edu.cn (H.Z.); zenglulu2025@wmu.edu.cn (L.Z.); zhongwei-zhao@wmu.edu.cn (Z.Z.); lixia2025@wmu.edu.cn (X.L.); 2111110079@bjmu.edu.cn (Q.W.); yangyang0502@wmu.edu.cn (Y.Y.); minjiangchen@wmu.edu.cn (M.C.); 2 Cixi Biomedical Research Institute, Wenzhou Medical University, Ningbo 315300, China; 3 Department of Radiology, Lishui Central Hospital, Lishui 323000, China; 4 Key Laboratory of Precision Medicine of Lishui, Lishui 323000, China; 5 Clinical College of The Affiliated Central Hospital, School of Medcine, Lishui University, Lishui, 323000, China

**Keywords:** PHF20L1, cancer, epigenetic regulation, histone methylation, targeted therapy

## Abstract

Plant homeodomain (PHD) finger protein 20-like 1 (PHF20L1) is a novel epigenetic “reader” that specifically recognises histone post-translational modifications (PTMs) via its Tudor and PHD finger domains, thereby regulating chromatin remodelling, DNA damage repair, and oncogene transcriptional activation. This review comprehensively summarises the role of PHF20L1 in various cancers, including breast, ovarian, and colorectal cancers, as well as retinoblastomas, and elucidates its molecular mechanisms of action in cancer pathogenesis. Accumulating evidence indicates that PHF20L1 is upregulated in these malignancies and drives tumour progression by promoting proliferation, metastasis, and immune evasion. Furthermore, PHF20L1 orchestrates tumour-related gene expression by interacting with key epigenetic complexes. Given its unique structural features, we propose novel strategies for developing small-molecule inhibitors and combinatorial therapies, providing a theoretical basis for targeted epigenetic regulation for precision treatment. Future research should further investigate the molecular regulatory networks of PHF20L1 in different cancers and other human diseases and focus on developing specific small-molecule inhibitors to enable precision-targeted therapies.

## 1. Introduction

Epigenetic dysregulation is a key driver of cancer development, promoting the malignant transformation and adaptive evolution of tumour cells by dynamically regulating chromatin modifications and gene expression programmes [1]. Histone modifications are crucial epigenetic mechanisms. Post-translational modifications (PTMs) of histone proteins, including methylation, acetylation, ubiquitination, phosphorylation, and SUMOylation, can alter chromatin accessibility, thereby regulating the recruitment and activity of the transcriptional machinery [2]. Aberrant histone modifications are key drivers of tumour cell proliferation, invasion, apoptosis, and stemness [3]. These chemical modifications are dynamic, reversible, and orchestrated by three major classes of epigenetic modifiers: writers, erasers, and readers. Writers, such as histone acetyltransferases (HATs) and histone methyltransferases (HMTs), catalyse the addition of specific modifications. Erasers, such as histone deacetylases (HDACs) and demethylases, remove these markers. In contrast, readers recognise and bind to specific histone modifications, serving as molecular interpreters that recruit downstream effector complexes to mediate transcriptional regulation and chromatin remodelling [4]. For instance, the plant homeodomain (PHD) finger domains can specifically recognise histone methylation marks [5]. Among these epigenetic regulators, reader proteins have gained increasing attention in recent years because of their central role in decoding histone modification signals and coordinating gene expression programmes [6]. For example, BRD4 recognises histone acetylation marks and promotes the transcription of multiple oncogenes, whereas HP1 binds to H3K9me3 to mediate heterochromatin formation and gene silencing [6,7]. Accumulating evidence suggests that dysregulation or reprogramming of epigenetic reader proteins is common across various cancers, positioning them as key nodes in the epigenetic network of tumour development and promising targets for therapeutic intervention.

**Figure 1:**
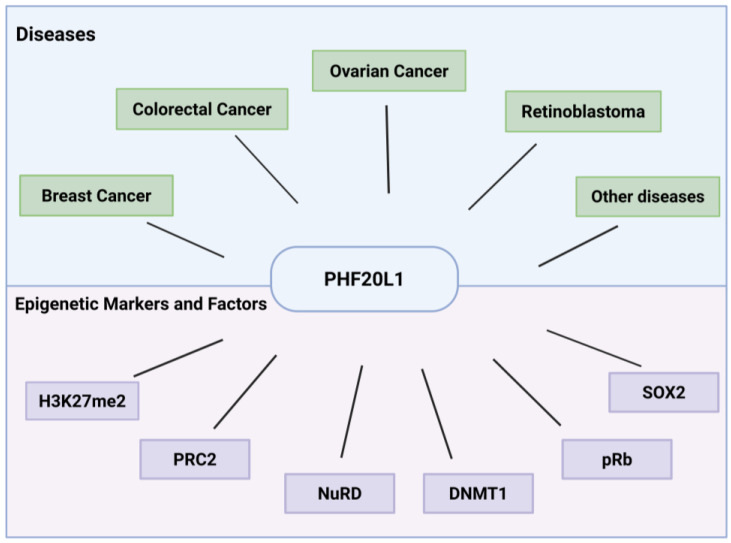
Versatile roles of PHF20L1 in cancer and epigenetic regulation. This schematic summarises PHF20L1’s oncogenic drivers in human cancers as well as its interactions with epigenetic markers and associated regulatory factors.

Plant homeodomain (PHD) finger protein 20-like 1 (PHF20L1) is a novel epigenetic reader that specifically recognises methylated histone marks (e.g., H3K27me2) [8] and non-histone proteins (e.g., SOX2) [9] through its unique Tudor and PHD finger domains. This recognition facilitates the recruitment of epigenetic regulatory complexes that reshape chromatin architecture and coordinate the transcriptional activation of oncogenes as well as the silencing of tumour suppressor genes. Recent studies have demonstrated that in addition to its physiological roles during normal development, PHF20L1 is aberrantly overexpressed in several malignancies, including breast [10], ovarian [11], and colorectal cancers [12]. Its overexpression contributes significantly to disease progression by promoting tumour cell proliferation, metastasis, immune evasion, and metabolic reprogramming (Figure 1).

## 2. Overview of PHF20L1

**Figure 2:**
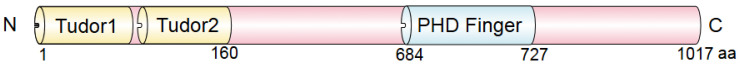
Domain-specific structural features of PHF20L1 underpin its epigenetic reader function. The structural architecture of PHF20L1, with a focus on its Tudor domains and PHD domain, reveals molecular mechanisms essential for mediating epigenetic regulation and driving cancer. The numbers indicate amino acid positions. Note: aa, amino acid.

PHF20L1, a protein that shares high homology with PHF20, functions as a methylated lysine reader and plays an important role in transcriptional regulation and chromatin remodelling. Both PHF20 and PHF20L1 are integral components of the non-specific lethal (NSL) complex and define two distinct NSL subcomplexes (PHF20-NSL and PHF20L1-NSL) with differential gene regulatory functions. The NSL complex is a multi-protein epigenetic regulatory complex. Its core components include the histone acetyltransferase MOF (KAT8), which catalyses the acetylation of histone H4 at lysine 16 (H4K16ac) and other sites (such as H4K5/K8) [13,14]. Both PHF20-NSL and PHF20L1-NSL complexes exert their gene regulatory functions by recruiting histone acetyltransferases (HAT) activity, thereby promoting transcriptional initiation [15,16]. Localised in both the nucleoplasm and cytoplasm, PHF20L1 is a multifunctional chromatin-binding protein with a unique structural architecture. As depicted in the diagram, the *N*-terminal of PHF20L1 features two Tudor domains, designated as Tudor1 and Tudor2 [17]. Additionally, a PHD finger domain located between amino acids 684 and 727 is present in the C-terminal region of the protein [18] (Figure 2). Collectively, these domains enable PHF20L1 to decode histone modification patterns, facilitate chromatin reorganisation, and modulate transcriptional regulation by dynamically engaging with various epigenetic factors.

**Function of the Tudor Domain**: The *N*-terminal of PHF20L1 contains two Tudor domains that can recognise and bind to methylated lysine residues, particularly those on histone and non-histone proteins [19]. This domain contains an aromatic cage, which is a binding pocket composed of aromatic amino acid residues. This cage can interact with the methylated lysine residues through π–π stacking interactions. This aromatic cage structure is crucial for PHF20L1’s recognition of methylated histones [20]. The Tudor domain of PHF20L1 interacts with various methylation sites. It can bind to the histone H3K27me2, participating in processes such as gene expression regulation, chromatin structure maintenance, and gene silencing [8]. In addition, PHF20L1 can bind to the monomethylated K142 site of DNA methyltransferase 1 (DNMT1) [21], thereby regulating the degradation of the DNMT1 protein and affecting the stability of DNA methylation.

**Function of the PHD Finger Domain**: The PHD domain recognises modifications at the terminal of histone H3, regulating gene transcription, chromatin structure, and various DNA-templated processes, such as DNA replication, recombination, and repair. These functions are crucial for maintaining genomic stability, regulating gene expression and responding to environmental changes [22,23]. The PHD domain is directly related to chromatin remodelling, nucleosome sliding, and DNA damage repair, mediating various nuclear signalling pathways required for the cell cycle, growth, and differentiation through the histone-binding function of a typical PHD finger, whereas the distortion of the PHD finger is associated with pathological development [24]. Studies have shown that the PHD domain of PHF20L1 plays a critical role in mouse development Mice lacking a functional PHD domain in PHF20L1 exhibit significant growth retardation and delayed mammary duct development [25]. However, the specific function and molecular mechanism of the PHD domain in PHF20L1 have not yet been reported and require further investigation.

**Histone Methylation Modification of PHF20L1**: Methylation is an essential post-translational histone modification. Histone methylation homeostasis, which is the balance between the addition and the removal of histone methylation modifications within cells, is crucial for maintaining gene expression, chromatin structure, and cellular functions. In humans, several histone methylation reader domains can recognise methylated or unmethylated lysine or arginine residues in histones. These proteins play crucial roles in epigenetic regulation of gene expression, chromatin structure, and DNA damage repair. Known readers typically contain PHD finger domains, WD40 repeats, colourless nonpigmented (CW) domains, proline–tryptophan–tryptophan–proline (PWWP) domains, and royal family domains, as well as proteins with chromatin, Tudor, and MBT repeats [26]. PHF20L1 contains a Tudor domain that is capable of recognising methylated histones. Tudor domain proteins (TDRDs) are mainly responsible for recognising methylated lysine and arginine residues [27,28,29]. The Tudor domain-containing protein PHF20L1 is a specific reader of histone H3K27me2, which recognises this modification and recruits PRC2 and the NuRD complex to regulate H3K27-related chromatin modifications and downstream gene expression [8]. H3K27me2 is one of the most abundant histone post-translational modifications in the host genome and is more widespread than H3K27 methylation [30]. The viral genome co-localises with the H3K27me2 reader protein PHF20L1, suggesting that PHF20L1 can recognise and bind to the viral genome associated with H3K27me2 modifications [31]. Moreover, this co-localisation is enhanced when H3K27 demethylases, Ubiquitously Transcribed Tetratricopeptide Repeat X-linked protein (UTX) and Jumonate Domain-Containing 3 (JMJD3), are inhibited, further indicating the presence of H3K27me2 associated with the viral genome and its interaction with PHF20L1, which may have important implications for viral gene expression and infection processes. PHF20L1 may be indirectly linked to the transcription-related functions of H3.3 through its involvement in transcriptional regulatory networks. PHF20L1 participates in the regulation of chromatin structure by recognising histone modifications and may play a role in transcriptionally active regions [32]. PHF20L1 interacts with various epigenetic complexes and regulates the expression of tumour-related genes through histone modifications [33]. H4K16ac is a histone modification that carries specific biological information. Males absent on the first (MOF), a catalytic subunit of the NSL complex, can acetylate multiple sites on histone H4 (such as H4K5, H4K8, and H4K16), and PHF20L1, as part of the NSL complex, may affect gene expression by regulating chromatin structure [33,34]. PHF20L1 regulates the levels of H4K16ac by recognising methylated sites and recruiting MOF complexes. This modification plays a key role in DNA damage response and may promote DNA repair by altering chromatin openness [18].

**Figure 3:**
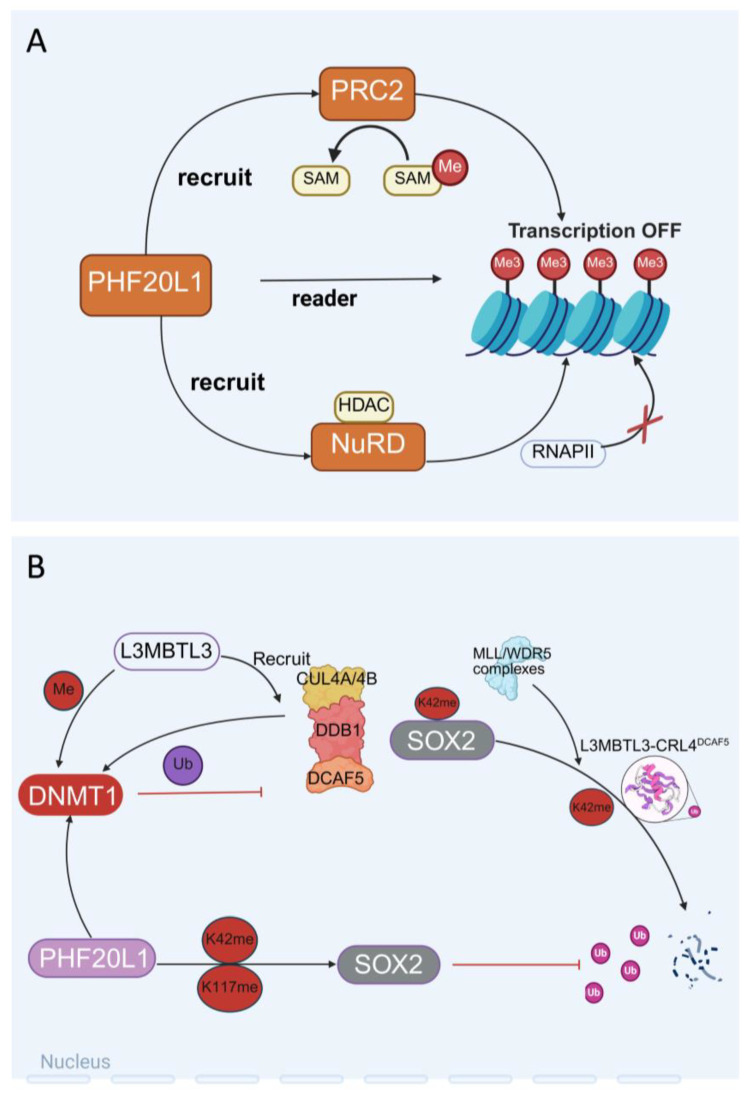
The dual role of PHF20L1 in epigenetic regulation and chromatin remodelling. (**A**) PHF20L1 recognises histone modifications and recruits epigenetic complexes to promote chromatin condensation and transcriptional repression. (**B**) The intricate protein interaction network within the nucleus, which includes L3MBTL3-mediated recruitment of the CUL4A/B-DDB1-DCAF5 complex to regulate DNMT1 localisation and activity, PHF20L1-dependent modulation of SOX2, and coordinated ubiquitination/methylation processes, demonstrates the pivotal role of PHF20L1 as a molecular orchestrator in nuclear protein dynamics.

A protein–protein interaction network was constructed using affinity purification and mass spectrometry, involving 11,464 interactions between 1738 different human proteins from 293 successfully purified proteins [35]. By comparing the networks of different cell lines, researchers have verified thousands of interactions and found extensive customisation between cell lines. Ultimately, these networks can be viewed interactively online using the BioPlexExplorer (BioPlex 3.0—HEK293T v3.0 & HCT116 v1.0, Boston, MA, USA) [36]. Similarly, the identification of PHF20L1’s interacting proteins using immunoprecipitation combined with mass spectrometry determined the interactions between PHF20L1 and Polycomb Repressive Complex 2 (PRC2) and nucleosome remodelling and deacetylation (NuRD) complexes. The methodological similarity between these approaches suggests that PHF20L1 research can draw on technical means to further explore its role in chromatin regulatory networks (Figure 3A).

**Non-histone Methylation Modification of PHF20L1**: PHF20L1 recognises histone methylation modifications and specifically binds to methylated lysine residues of non-histone proteins through its *N*-terminal tandem Tudor domains [18,21]. As a protein containing the PHD finger domain, the function of PHF20L1 is closely related to its conformational dynamics. Studies have shown that the first Tudor domain (Tudor1) of PHF20L1 is usually in a closed conformation in its free state. However, nuclear magnetic resonance (NMR) relaxation dispersion and molecular dynamics simulations have found that this domain has a low-abundance open conformation. In this conformation, the aromatic cage residues of Tudor1 undergo significant rearrangement, thereby specifically recognising the monomethylated modification at position K142 of DNMT1 [20]. DNMT1 is a key enzyme for maintaining the DNA methylation status, and its regulation is crucial for gene expression and cellular function. It is worth noting that K142 methylation of DNMT1 is catalysed by the SET domain-containing 7 (SET7) methyltransferase, and this modification can recruit L3MBTL3 and CRL4DCAF5 ubiquitin ligase complexes, mediating the ubiquitination and degradation of DNMT1 [37]. The L3MBTL3–CRL4DCAF5 complex targets the stability of methylated non-histone proteins (such as SOX2, DNMT1, and E2F1) by recognising the K142 methylation of DNMT1 and triggering its degradation [38]. Further studies have shown that PHF20L1 and Lysine-specific demethylase 1 (LSD1) work together during the S phase to counteract the degradation of DNMT1 by the L3MBTL3–CRL4DCAF5 complex, maintaining methylation homeostasis during DNA replication [39].

PHF20L1 also plays an important role in maintaining the stemness of tumour stem cells. SRY-box transcription factor 2 (SOX2), a core transcription factor that maintains the pluripotency of embryonic stem cells (ESCs), is closely associated with the acquisition of tumour stem cell-like characteristics and metastasis when its expression is dysregulated [40]. As a key factor, it plays a central role in maintaining the self-renewal and pluripotency of embryonic stem cells and adult stem cells. Overexpression of SOX2 is associated with the acquisition of stem cell-like characteristics by tumour cells, which may promote tumour initiation, invasion, and metastasis; hence, overexpression of the *SOX2* gene or protein can be observed in cancers [41,42,43]. PHF20L1 recognises the monomethylated sites K42 and K117 of SOX2 through its MBT domain, inhibiting ubiquitination and degradation of SOX2 mediated by the MLL1/WDR5 complex, thereby stabilising the protein level of SOX2 [9]. Experiments have confirmed that knockdown of PHF20L1 or LSD1 in PA-1 cells and mouse ESCs leads to the loss of SOX2 protein stability and significantly impairs the self-renewal capacity of stem cells [44,45,46], which provides a new molecular basis for therapeutic strategies targeting *SOX2*-positive tumours (Figure 3B).

## 3. The Role of PHF20L1 in Cancer

**Figure 4:**
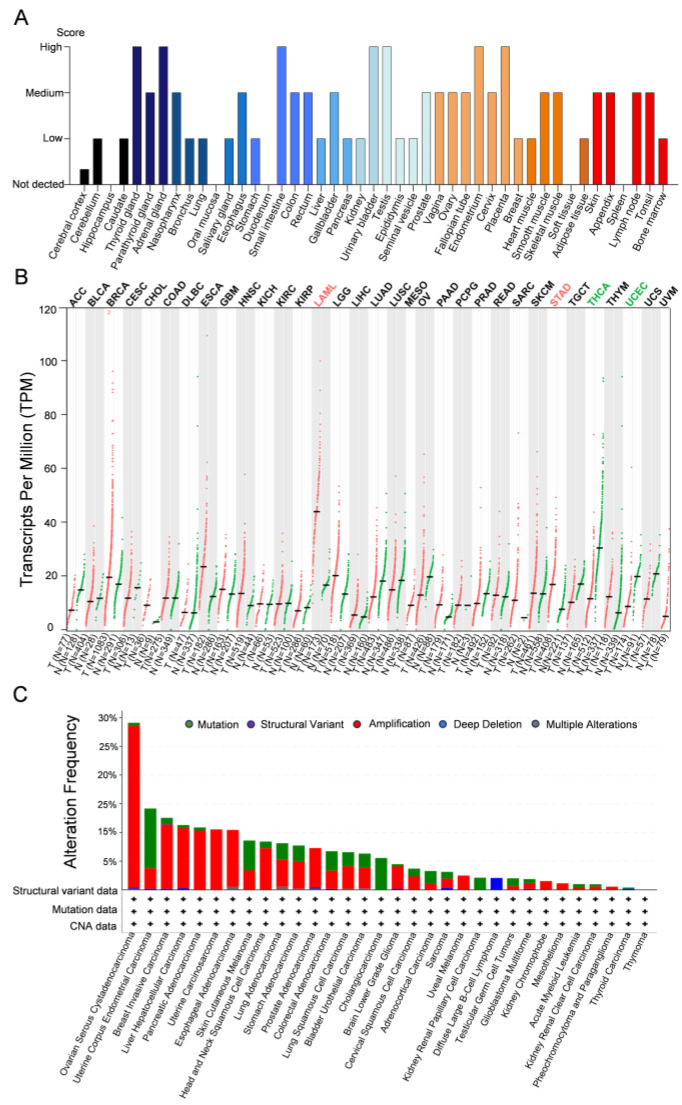
Comparative analysis of PHF20L1 expression in cancers and normal tissues. (**A**) The distribution of PHF20L1 expression levels across various tissues, highlighting its expression intensity in normal tissues. (**B**) PHF20L1 gene expression levels across various cancer types (measured in transcripts per million, TPM). Red and green dots represent PHF20L1 expression in tumour and normal tissues, respectively; error bars show standard deviation. (**C**) The alteration frequency of PHF20L1 in various cancers, based on TCGA data, includes mutations (green, point mutations or small insertions/deletions), structural variants (purple, large-scale chromosomal rearrangements), amplifications (red, increase in gene copy number), and deep deletions (blue, homozygous loss of both gene copies). The data presented in the figure were obtained from open-access platforms and visualised, including the Human Protein Atlas (https://www.proteinatlas.org/), GEPIA 2 (http://gepia2.cancer-pku.cn/#index, accessed on 25 April 2025), and cBioPortal (https://www.cbioportal.org/). All data were used in accordance with the terms of use specified by each database for academic and non-commercial purposes.

PHF20L1 is expressed in various tissue, both in normal physiological contexts and in pathological conditions, such as cancer. Analysis of data from the Human Protein Atlas (https://www.proteinatlas.org/) [47,48] indicated that PHF20L1 is highly expressed in the thyroid gland, adrenal gland, small intestine, urinary bladder, testis, endometrium, and placenta, suggesting that PHF20L1 may have tissue-specific expression patterns (Figure 4A). Analysis of the Cancer Genome Atlas (TCGA) database using the Gene Expression Profiling Interactive Analysis platform GEPIA 2 (http://gepia2.cancer-pku.cn/, accessed on 25 April 2025) [49] revealed that in some cancer types, such as acute myeloid leukaemia (LAML) and oesophageal adenocarcinoma (ESCA), the expression levels of PHF20L1 in tumour tissues were significantly higher than those in normal tissues. This suggested that PHF20L1 plays an important role in the development and occurrence of these cancers. However, in other cancer types, such as Uterine Corpus Endometrial Carcinoma (UCEC) and thyroid carcinoma (THCA), the expression levels of PHF20L1 were downregulated in tumour tissues compared to those in normal tissues, which suggests that the role of PHF20L1 in these cancers may have an opposite effect (Figure 4B). In recent years, research on PHF20L1 has focused mainly on various malignant tumours, including breast, ovarian, and colorectal cancers, which accelerate the malignant development of cancer by promoting tumour cell proliferation, metastasis, and immune evasion. In addition, analysis of TCGA using cBioPortal for Cancer Genomics (http://www.cbioportal.org) [50] revealed that PHF20L1 exhibits diverse alteration frequencies and types across multiple cancers. Notably, PHF20L1 was highly amplified in several cancers, including ovarian serous cystadenocarcinoma, breast cancer, liver cancer, and pancreatic adenocarcinoma, suggesting that it may play a critical role in tumourigenesis (Figure 4C). Moreover, PHF20L1 alterations not only affect its own function but may also affect interactions with other genes, further influencing tumour cell proliferation, apoptosis, invasion, and metastasis.

**Breast Cancer**: The role of PHF20L1 in mouse development has been studied extensively. Its absence caused growth retardation in mice and delayed the development of mammary ducts [25]. In breast cancer, the expression of PHF20L1 was elevated and positively correlated with histological grading. Immunohistochemical (IHC) analysis further revealed that the expression of PHF20L1, enhancer of zeste homologue 2 (EZH2), and methylthiotransferase 1 (MTA1) were simultaneously upregulated and positively correlated with histological grading. A comprehensive analysis integrating copy number variations, gene expression, clinicopathological features, and patient survival data for 41 *TDRD* genes in breast cancer revealed that PHF20L1 was the most prominently amplified *TDRD* gene in TCGA breast cancer samples. Amplification and overexpression of PHF20L1 were more significant in aggressive basal-like and Luminal B subtypes of breast cancer. The methylation level of the PHF20L1 promoter region is positively correlated with breast cancer metastasis [17]. PHF20L1 is an H3K27me2 recognition protein characterised by the Tudor domain and is a potential *MYC*- and hypoxia-driven oncogene that can recruit PRC2 and NuRD to specific chromatin regions, exerting transcriptional repression activity. The main function of PRC2 is to catalyse H3K27me3, a modification associated with gene silencing. The NuRD complex is responsible for the H3K27 deacetylation, a modification that makes the chromatin more compact, thereby inhibiting gene expression. PHF20L1 enhances the Warburg effect by promoting the methylation and deacetylation of H3K27, thereby promoting tumour progression. PHF20L1 directly inhibits a series of tumour suppressor factors, including Forkhead Box Protein K2 *(FOXK2)* and Hypermethylated in Cancer 1 (*HIC1*), through its synergistic action with the PRC2 and NuRD complexes, thereby driving glycolysis and promoting tumour occurrence [8].

In breast cancer, the expression of PHF20L1 may be regulated by microRNAs. MiR-96-5p, miR-9-5p, and miR-182-5p may target PHF20L1 directly or indirectly, regulate genes involved in selective splicing, and contribute to tumour occurrence, invasion, and metastasis [10]. MicroRNAs (miRNAs) are short non-coding RNA molecules, 19–24 nucleotides in length, which do not encode proteins in cells. MiRNAs can regulate multiple genes in the same biological pathway. miR-96-5p is thought to be involved in EMT. miR-9-5p can enhance the cancer stem cell-like characteristics of breast cancer and may promote breast cancer metastasis by enhancing cancer stem cell properties [51]. These miRNAs may play a significant role in the expression changes in PHF20L1, thereby promoting its role in breast cancer metastasis.

**Ovarian Cancer**: The tumour microenvironment (TME) consists of various cells, including fibroblasts, endothelial cells, and immune cells (such as T and B cells), which interact by secreting cytokines and growth factors that affect tumour growth and metastasis. Ascites, an important component of the ovarian cancer microenvironment, contains various cytokines, growth factors, and extracellular matrix compounds [52,53]. Studies have shown that ascites can affect the expression and function of PHF20L1 isoforms by altering various compounds in the ovarian cancer microenvironment, thereby participating in the regulation of the biological behaviour of ovarian cancer cells, including fucosylation, cell adhesion, tumour growth, and drug resistance [54]. PHF20L1 is expressed as a fucosylated protein in ovarian cancer tissues and its expression increases in SKOV-3 cells stimulated by ascites in patients with ovarian cancer [55]. Immunohistochemical findings indicated that PHF20L1 was overexpressed in tumour tissue sections obtained from patients with ovarian cancer, and elevated PHF20L1 expression was associated with reduced progression-free and overall survival. Furthermore, Western blotting analyses revealed that protein isoforms were differentially regulated in SKOV-3 cells upon stimulation with ascites in patients with epithelial ovarian cancer [11].

**Colorectal Cancer**: The TCGA database showed that PHF20L1 is highly expressed in colorectal cancer tissues and is closely related to tumour growth and progression. PHF20L1 promotes EMT of colorectal cancer cells and increases their invasiveness and metastatic abilities; it also regulates the expression of various angiogenic factors (such as ANGPT2, FGF1, PDGFA, and VEGFA) and promotes angiogenesis in colorectal cancer tissues. Angiogenesis provides a rich blood supply to tumours, supporting their rapid growth and distant metastasis. PHF20L1 inhibits the expression of *HIC1*, indirectly promoting the expression of Paired Box Gene 2 (*PAX2*) and driving malignant progression of colorectal cancer cells. PHF20L1 not only plays an important role in the primary tumour but also promotes liver metastasis of colorectal cancer cells through the aforementioned mechanisms (such as promoting EMT and angiogenesis), leading to poor prognosis [12].

**Retinoblastoma**: Interaction between PHF20L1 and monomethylated pRb is crucial for maintaining the integrity of the pRb-dependent G1-S checkpoint. The G1-S checkpoint is an important checkpoint in the cell cycle that ensures that various conditions within the cell, including DNA integrity, are suitable before entering the S phase (DNA synthesis phase) from the G1 phase. If this checkpoint is abnormal, cells may enter the S phase without repairing DNA damage, causing genomic instability and increasing the risk of cancer. PHF20L1 can read the monomethylation of K810 on phosphorylated retinoblastoma tumour suppressor protein (pRb), effectively integrating pRb activity with the DNA damage response [18].

**Figure 5:**
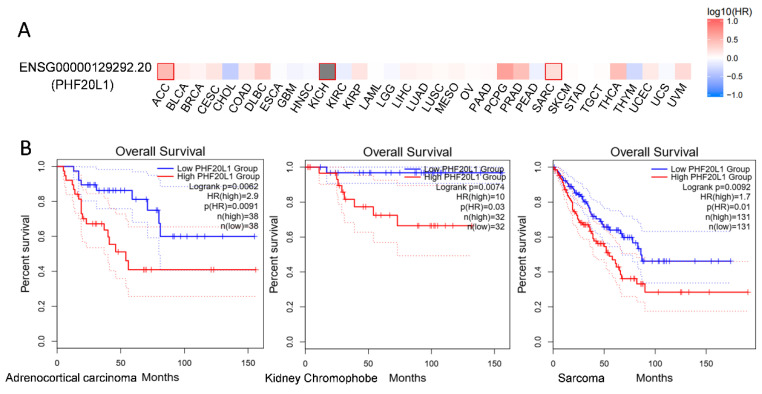
Expression and survival analysis of PHF20L1 in various cancers based on TCGA data. (**A**) PHF20L1 exhibited diverse expression patterns across various cancers and was significantly associated with overall patient survival. Heatmap illustrates PHF20L1 expression in different cancers, with red indicating high expression and blue indicating low expression. (**B**) The TCGA database revealed a notable correlation between PHF20L1 expression and overall patient survival. In sarcomas, adrenocortical carcinomas, and thyroid carcinomas, patients with high PHF20L1 expression showed markedly worse survival rates. The experimental data displayed in this figure were sourced from publicly accessible databases, including GEPIA 2 (http://gepia2.cancer-pku.cn/#index, accessed on 25 April 2025), in full compliance with the data usage policies and terms of service of the database.

**Other Cancers**: In addition to the above cancers, PHF20L1 has been identified as a prognostic biomarker for colorectal cancer (CRC) by multivariate Cox regression. Patients with high-risk scores had a worse overall survival (OS) than those with low-risk scores [56]. Further analysis using the GEPIA 2 database (http://gepia2.cancer-pku.cn/, accessed on 25 April 2025) revealed that PHF20L1 showed significant prognostic correlation with kidney chromophobes (KICH), sarcoma (SARC), and adrenocortical carcinoma (ACC) (Figure 5A). Survival analysis showed that the OS of patients in the high PHF20L1 expression group was significantly lower than that of patients in the low PHF20L1 expression group. Specifically, in sarcoma, the hazard ratio (HR) is 1.7 (*p* = 0.0092); in adrenocortical carcinoma, the HR is 2.9 (*p* = 0.0091); and in kidney chromophobes, the HR is 10 (*p* = 0.0074) (Figure 5B). Therefore, future research should combine tissue-specific functions and utilise multi-omics technologies, single-cell sequencing, and protein interaction network analysis to explore the molecular regulatory networks of PHF20L1 in different cancers and reveal its key roles in tumour occurrence and development.

## 4. Targeting PHF20L1’s Epigenetic Domains to Develop Cancer Therapeutics

The oncogenic role of PHF20L1 in cancer and its epigenetic regulatory characteristics make it a potential therapeutic target, particularly for cancers with overexpression and poor prognosis. Targeting its functions or developing combined therapeutic strategies may provide new directions for cancer treatment.

**Inhibition Strategies Targeting the Tudor Domain**: The Tudor domain of PHF20L1 recognises methylated lysine residues through conformational selection, suggesting that it can be targeted by small-molecule inhibitors [20], making it a druggable target. High-throughput screening strategies based on biotin-labelled compound libraries not only distinguished compounds selectively binding to the Tudor domain of PHF20 but also identified a compound with binding affinity for the Tudor domain of PHF20L1 and other domains containing an “aromatic cage”. Structural optimisation studies have identified SPIN1-selective inhibitors that are active in cell-based assays [57]. These studies provide an important foundation for the future development of inhibitors targeting the Tudor domain of PHF20L1.

**Regulatory Strategies Targeting the DNMT1 Interaction Network**: PHF20L1 regulates the stability of DNMT1 by directly interacting with it, thereby affecting DNA methylation status and gene expression. This mechanism plays an important role in the maintenance of cancer stem cells and in chemotherapeutic resistance. Targeting PHF20L1 or its related pathways, such as combining DNMT1 and G9a/GLP inhibitors, may be an effective strategy to reverse chemotherapeutic resistance and enhance the effectiveness of cancer treatments. Combined inhibitors of DNMT1 (e.g., decitabine) and G9a/GLP (e.g., UNC0638) can synergistically reverse chemotherapeutic resistance, reduce DNA methylation, and restore the expression of genes silenced by abnormal methylation [58]. Notably, DNMT1/HDAC dual inhibitors, such as the novel compound (R)-23a, has shown significant antitumour activity in solid tumour models by simultaneously inhibiting DNA methylation and histone deacetylation [59]. Additionally, mitomycin A can block the PHF20L1-DNA interaction by competitively binding to GC-rich DNA sequences, while HDAC inhibitors (entinostat and trichostatin A) weaken the affinity of PHF20L1 for methylated histones by inducing histone hyperacetylation.

**Targeting the PHD Finger Domain and Downstream Signalling Pathways**: The PHD finger is a key effector of histone post-translational modifications and acts as a regulator of gene expression and genomic integrity, dysfunction of PHD finger-containing proteins is associated with various human diseases. The role of PHF20L1 in multiple cancers suggests that PHD inhibitors may inhibit the function of PHF20L1 by modulating the PHD fingers’ activity, thereby slowing tumour progression. Moreover, PHF20L1 promotes tumour metabolic reprogramming, such as the Warburg effect, and chemotherapeutic resistance by regulating the MYC/HIF-1α signalling axis [60]. Small-molecule inhibitors, such as 10058-F4, inhibit transcriptional activity by disrupting the formation of MYC-MAX dimers and reducing HIF-1α expression and inducing cell cycle arrest/apoptosis in colorectal and ovarian cancer models [61,62].

**Immunotherapy Combination Strategies**: As a component of the NSL complex, PHF20L1 regulates immune responses in tumour microenvironment (TME) by coordinating histone acetylation, including HDAC-mediated chromatin remodelling [63]. HDAC inhibitors (TSA, entinostat) can upregulate immune-related genes, such as those in the interferon pathway and enhance antigen presentation, thereby promoting T-cell infiltration and killing [64,65,66]. Preclinical studies have shown that TSA-modified tumour vaccines can activate innate/adaptive immune responses and significantly inhibit the progression of ovarian cancer [67]. Combining PHF20L1-targeted therapy with immune checkpoint inhibitors may produce synergistic antitumour effects.

Currently, research on the mechanism of action of PHF20L1 remains at an exploratory stage. Future studies should integrate multi-omics data, such as single-cell sequencing and protein interaction networks, to elucidate molecular regulatory networks across different cancer types. Specific small-molecule inhibitors have been developed based on their domain characteristics (e.g., Tudor or PHD finger) to achieve precision in oncology and enhance therapeutic specificity.

## 5. Exploring PHF20L1 in Other Disease Contexts

The chromosomal region 8p23.1 overlaps with a gene cluster regulating natural killer (NK)-cell activity, suggesting that PHF20L1 participates in pathogenesis by coordinating innate and adaptive immune responses. However, current evidence is mainly based on statistical associations and functional experiments, such as CRISPR-Cas9-mediated gene editing or humanised mouse models, which are needed to verify the direct impact of PHF20L1 variants on the autoimmune attack of pancreatic β cells and T-cell differentiation [68]. Although the current research has mainly focused on the function of PHF20L1 in cancer, its pleiotropic effects may extend to other diseases or physiological processes. As a multidomain epigenetic regulatory protein, PHF20L1 may contribute to diverse pathological processes by regulating key genes and signalling pathways. Its role in maintaining SOX2 stability may be implicated in neurodevelopmental disorders, whereas abnormalities in epigenetic regulation, such as the dysregulation of H3K27 methylation, may be linked to neurodegenerative diseases. The activating pathways, such as MYC, that influence metabolic reprogramming may contribute to obesity or diabetes. Interactions between PRC2/NuRD complexes may regulate inflammation-related genes and indirectly affect autoimmune diseases. As a member of the Tudor domain protein family, it may play a role in abnormal germ cell development and infertility. Future studies should investigate these mechanisms to clarify their disease-specific roles and therapeutic potential.

Our search using the publicly accessible human disease gene database platform DisGeNET (https://disgenet.com/) [69,70] revealed that PHF20L1 was potentially associated with diseases other than cancer (Table 1). In addition to various cancers, we identified several non-tumour diseases including sickle-cell anaemia, liver secondary malignant tumours, and goitres. This further suggests the potential contribution of PHF20L1 in multiple diseases; however, further validation is needed.

## 6. Conclusions and Prospects

PHF20L1 is a protein with Tudor and PHD finger domains that plays a pivotal role in the epigenetic regulation of cancer progression and other diseases. Its unique structure enables the recognition of histone modifications, thereby influencing chromatin remodelling and gene expression. PHF20L1 has been confirmed to influence malignant tumours and is a promising biomarker for diagnosis and treatment. Its overexpression in various cancers facilitates tumour growth, metastasis, and immune evasion through interactions with epigenetic complexes. It also plays a role in non-oncogenic diseases, indicating its diverse biological functions. Future research should aim to systematically dissect the signalling networks and epigenetic landscapes involving PHF20L1. Investigating its role in tumour microenvironment remodelling, drug resistance, and immunoregulation may open new avenues for cancer therapy. Additionally, given its potential as a chromatin-binding therapeutic target, the development of small-molecule inhibitors or degraders targeting PHF20L1 may hold promise as a precision medicine strategy in oncology.

## Figures and Tables

**Table 1:** Diseases associated with PHF20L1 identified from the DisGeNET database.

NO	Disease	Disease Class	Semantic Type	References
1	Carcinoma of Breast	Neoplasms, Skin and Connective Tissue Diseases	Neoplastic Process	[71,72,73]
2	Ascites	Pathological Conditions, Signs and Symptoms	Disease or Syndrome	[74,75]
3	Breast Neoplasms	Skin and Connective Tissue Diseases, Neoplasms	Neoplastic Process	[76,77,78,79]
4	Cancer, Breast	Skin and Connective Tissue Diseases, Neoplasms	Neoplastic Process	[80]
5	Colorectal Neoplasm	Digestive System Diseases, Neoplasms	Neoplastic Process	[81]
6	Anaemia, Sickle Cell	Congenital, Hereditary, and Neonatal Diseases and Abnormalities, Hemic and Lymphatic Diseases	Disease or Syndrome	[82]
7	Secondary malignancy of liver	Neoplasms, Digestive System Diseases	Neoplastic Process	[83,84,85]
8	Carcinogenesis	Neoplasms, Pathological Conditions, Signs and Symptoms	Neoplastic Process	[86]
9	CRC	Digestive System Diseases, Neoplasms	Neoplastic Process	[87,88]
10	Cancer, Ovarian	Urogenital Diseases, Neoplasms, Endocrine System Diseases	Neoplastic Process	[89,90]
11	Colorectal Cancer		Neoplastic Process	[91]
12	Malignant Neoplasm of Brain	Neoplasms, Nervous System Diseases	Neoplastic Process	[92,93,94]
13	Luminal B Breast Carcinoma		Neoplastic Process	[95,96,97]
14	Neoplasm, Ovarian	Urogenital Diseases, Neoplasms, Endocrine System Diseases	Neoplastic Process	[89,98]
15	Mathematical ability	Psychological Phenomena	Mental Process	[99]
16	Percentage of body fat		Finding	[100,101]
17	Goitre	Endocrine System Diseases	Disease or Syndrome	[102]
18	Educational Achievements		Finding	[103]
19	Q-T interval, NOS		Clinical Attribute	[104]
20	Body Height		Organism Attribute	[105]

## Data Availability

All data required to evaluate the conclusions are presented in this paper. The expression of PHF20L1 in different cancers was downloaded from the Human Protein Atlas (https://www.proteinatlas.org/ENSG00000129292-PHF20L1/tissue, accessed on 25 April 2025) and the Gene Expression Profiling Interactive Analysis platform GEPIA 2 (http://gepia2.cancer-pku.cn/#general, accessed on 25 April 2025). The alteration frequency of PHF20L1 in various cancers can be downloaded from the cBioPortal For Cancer Genomics (https://www.cbioportal.org/results/cancerTypesSummary?case_set_id=all&gene_list=PHF20L1&cancer_study_list=5c8a7d55e4b046111fee2296, accessed on 25 April 2025). The association between PHF20L1 with human diseases other than cancer was downloaded from the publicly accessible human disease gene database platform DisGeNET (https://disgenet.com/search?view=GENES&idents=5, accessed on 25 April 2025).

## Abbreviations

The following abbreviations are used in this manuscript:

ACC	Adrenocortical carcinoma
BLCA	Bladder urothelial carcinoma
BRCA	Breast invasive carcinoma
CESC	Cervical squamous cell carcinoma
CRC	Colorectal cancer
CHOL	Cholangiocarcinoma
COAD	Colon adenocarcinoma
CW	Colourless non-pigmented
DNMT1	DNA methyltransferase-1
EMT	Epithelial–mesenchymal transition
ESCA	Esophageal carcinoma
ESCs	Embryonic stem cells
EZH2	Enhancer of Zeste Homologue 2
FOXK2	Fork head box protein K2
GBM	Glioblastoma multiforme
HAT	Histone acetyltransferase activity
HDACs	Histone deacetylases
HMTs	Histone methyltransferases
HIC1	Hypermethylated in Cancer 1
H3K4me3	Trimethylation of lysine 27 on histone H3
H3K27me2	Di-methylation of lysine 27 on histone H3
H3K27me3	Tri-methylation of lysine 27 on histone H3
H4K16ac	H4 “Lys-16” acetylation
HNSC	Head and neck squamous cell carcinoma
IHC	Immunohistochemical
JMJD3	Jumonate Domain-Containing 3
KICH	Kidney chromophobe
KIRC	Kidney renal clear cell carcinoma
KIRP	Kidney renal papillary cell carcinoma
LAML	Acute myeloid leukaemialeukemia
LGG	Brain lower grade glioma
LIHC	Liver hepatocellular carcinoma
LSD1	Lysine-specific demethylase 1
LUAD	Lung adenocarcinoma
LUSC	Lung squamous cell carcinoma
MBT	Malignant brain tumour
MESO	Mesothelioma
MOF	Males absent on the first
MTA1	Methylthiotransferase 1
MYC	MYC Proto-Oncogene
NMR	Nuclear magnetic resonance
NSCLC	Non-small cell lung cancer
NuRD	NucleCome Remodelling and Deacetylase complex
NSL	Non-specific lethal
OS	Overall survival
OV	Ovarian serous cystadenocarcinoma
PAAD	Prostate adenocarcinoma
PAX2	Paired Box Gene 2
PWWP	Proline–tryptophan–-tryptophan–-proline
PHD	Plant homeodomain
PHF20	PHD finger protein 20
PHF20L1	PHD finger protein 20-like protein 1
PRAD	Prostate adenocarcinoma
PRC2	Polycomb Repressive Complex 2
PTMs	Post-translational modifications
READ	Rectum adenocarcinoma
SARC	Sarcoma
SET7	SET Domain Containing 7
SKCM	Skin cutaneous melanoma
STAD	Stomach adenocarcinoma
TCGA	The Cancer Genome Atlas
TGCT	Testicular germ cell tumours
THCA	Thyroid carcinoma
THYM	Thymoma
TME	Tumour microenvironment
UCEC	Uterine corpus endometrial carcinoma
UCS	Uterine carcinosarcoma
UTX	Ubiquitously Transcribed Tetratricopeptide Repeat X-linked protein
UVM	Uveal melanoma
